# Dr. Bryce’s Method of Collecting

**Published:** 1909-07

**Authors:** 


### Dr. Bryce's Method of Collecting.

---

Dr. C. A. Bryce is editor and publisher of the *Southern Clinic*, of Richmond, Va. He has worked out a method of collecting physicians' bills. With Dr. Bryce's consent we present the plan in full below. We will introduce the plan in the Doctor's own words, and he is welcome to the free advertising that this involves.

BRYCE'S COLLECTOR.

Dear Doctor: We know from experience that doctors are the poorest men in the world, and it is because they are the poorest business men on earth today. You are keeping yourself out of hundreds of dollars annually that you could easily get without offending your patients or without any worry on your part.

We have solved the problem in our system of statements.

It has worked so well for us that we are supplying the profession. Here is our guarantee: If you will commence the coming year with our system, and carry it out with every one of your patients, regardless of how good or how worthless they may be, we will cheerfully refund the money you pay us if you do not admit that we have made you better off financially than you have been for many years.

We furnish you with 500 bills, assorted—about 165 of a kind—for $1.00, and send you *The Southern Clinic* for a year.

Now, our method looks extremely simple, but it is the best system ever devised by any individual or agency, so far as our experience goes. On the first of each month fill out bills No. 1 and mail to each and every one of your debtors. Don't leave them in person —mail them. You have no idea how much virtue there is in a letter or bill under seal. Next month send No. 2 to all. Third month send No. 3.

This will settle every bill you have out owing you by people worth attending. Those who do not respond to these bills should be refused any further attention. Try the system, Doctor, you will thank us. We can print your name on the bills for 25 cents extra, but think it better to write the name, making it appear that others beside you are trying to get their fees.

Fraternally yours,

SOUTHERN CLINIC.

4 E. Clay St., Richmond, Va.

Exact copies of the bills, Nos. 1, 2 and 3, follow:

When you send for a doctor it is because you need him and expect him to come. When he sends you his bill it is because he needs your assistance and expects it.

MR. ............................................

*To Dr.* ........................ *Dr.*

*To Professional Services to date.* $.............

*Date* ............................

If you send for a doctor twice in succession you would be in great need of him and would think very badly of him if he neglected you under such conditions. This is your second bill.

MR. ............................................

*To Dr.* ........................ *Dr.*

*To Professional Services to date.* $.............

*Date* ............................

When a drowning man goes down for the third time his friends have lost their chances to save him. This is our third appeal to you to treat us fairly. Don't drown a good friend.

MR. ............................................

*To Dr.* ........................ *Dr.*

*To Professional Services to date.* $.............

*Date* ............................

—*From Medical World, June.*

